# Human Bone Derived Collagen for the Development of an Artificial Corneal Endothelial Graft. *In Vivo* Results in a Rabbit Model

**DOI:** 10.1371/journal.pone.0167578

**Published:** 2016-12-01

**Authors:** Natalia Vázquez, Manuel Chacón, Carlos A. Rodríguez-Barrientos, Jesús Merayo-Lloves, Miguel Naveiras, Begoña Baamonde, Jose F. Alfonso, Iriana Zambrano-Andazol, Ana C. Riestra, Álvaro Meana

**Affiliations:** 1 Instituto Universitario Fernández-Vega. Universidad de Oviedo (Spain); 2 Hospital Universitario Central de Asturias (Spain); 3 CIBER on rare disease–CIBERer. Instituto de Investigaciones Sanitarias de la Fundación Jiménez Díaz–IIS-FJD (Spain); Cedars-Sinai Medical Center, UNITED STATES

## Abstract

Corneal keratoplasty (penetrating or lamellar) using cadaveric human tissue, is nowadays the main treatment for corneal endotelial dysfunctions. However, there is a worldwide shortage of donor corneas available for transplantation and about 53% of the world’s population have no access to corneal transplantation. Generating a complete cornea by tissue engineering is still a tough goal, but an endothelial lamellar graft might be an easier task. In this study, we developed a tissue engineered corneal endothelium by culturing human corneal endothelial cells on a human purified type I collagen membrane. Human corneal endothelial cells were cultured from corneal rims after corneal penetrating keratoplasty and type I collagen was isolated from remnant cancellous bone chips. Isolated type I collagen was analyzed by western blot, liquid chromatography -mass spectrometry and quantified using the exponentially modified protein abundance index. Later on, collagen solution was casted at room temperature obtaining an optically transparent and mechanically manageable membrane that supports the growth of human and rabbit corneal endothelial cells which expressed characteristic markers of corneal endothelium: zonula ocluddens-1 and Na^+^/K^+^ ATPase. To evaluate the therapeutic efficiency of our artificial endothelial grafts, human purified type I collagen membranes cultured with rabbit corneal endothelial cells were transplanted in New Zealand white rabbits that were kept under a minimal immunosuppression regimen. Transplanted corneas maintained transparency for as long as 6 weeks without obvious edema or immune rejection and maintaining the same endothelial markers that in a healthy cornea. In conclusion, it is possible to develop an artificial human corneal endothelial graft using remnant tissues that are not employed in transplant procedures. This artificial endothelial graft can restore the integrality of corneal endothelium in an experimental model of endothelial dysfunction. This strategy could supply extra endothelial tissue and compensate the deficit of cadaveric grafts for corneal endothelial transplantation.

## Introduction

Corneal transplantation is the main treatment for patients suffering corneal endothelial dysfunctions. Nevertheless, an important problem in several countries of the world is the shortage of donors, and about 53% of the world’s population have no access to corneal transplantation [1]. New therapeutic approaches have been appeared in recent years. Medical treatment with ROCK kinase inhibitor [2] and cell therapies associated with ROCK kinase inhibitor [3] are currently being evaluated. Despite the promise of these new therapeutics, keratoplasty from corneal cadaveric donors remains the gold standard for endothelial diseases.

Penetrating keratoplasty (PK) has traditionally been the treatment of choice for eyes with a damaged endothelial layer such as in Fuchs’ endothelial dystrophy or in pseudophakic bullous keratopathy. However, there are several drawbacks with regard to PK such as graft rejection, suture-related problems, infection and astigmatism. New surgical options, such as Descemet’s membrane endothelial keratoplasty (DMEK) or Descemet’s striping automated endothelial keratoplasty (DSAEK), have become increasingly popular since they tend to optimize corneal resources by replacing only the damaged part of the cornea [4–7]. These new techniques require even better endothelial quality in order to perform the corneal graft (cell density, hexagonality ratio, etc), so only 30–35% of the corneas are suitable for lamellar endothelial keratoplasty [8].

A DMEK graft consists of a monolayer of corneal endothelial cells (CECs) on its Descemet´s membrane. Reproducing this structure by tissue engineering techniques requires a scaffold that mimics the Descemet´s membrane and a source of cells capable of restoring the endothelial function, reflecting two critical determinants for a successful tissue engineered product: the *in vitro* culture of human CECs and the development of a scaffold that provides the appropriate environment for cells.

Human CECs are considered as non-proliferative *in vivo*, since they are arrested in G1 phase [9,10]. Therefore the number of human CECs decreases with age [11] and in several diseases such as endothelial dystrophy, glaucoma, or cataract surgery [12,13]. On the other hand, several studies have shown that human CECs can be induced to divide to a limited extent *in vitro* [14] so that expansion of cultured human CECs could potentially allow many patients to be treated using one donor, decreasing some of the current donor shortage problems.

Different carriers have been used as scaffold for endothelial tissue engineering such as denuded Descemet´s membrane [15,16], amniotic membrane [17], gelatin membrane [18–21], anterior lens capsule [22], silk fibroin membrane [23], and different synthetic polymers [24]. In recent years, several groups have been using natural [25–27] or recombinant [28] collagen scaffolds for the culture of human CECs, being porcine [29] or bovine [30,31] type I collagen the most widely used.

Type I collagen is the most abundant protein constituting approximately 25 to 30% of all proteins of human body [32]. It is also an important component of all connective tissues of the body: muscle, teeth, bone and skin [33]. A natural source of type I collagen is cancellous bone [34]. Human cancellous bone is the second most processed, distributed and grafted tissue worldwide, coming right after blood transfusion [35]. The remnant cancellous bone generated during the preparation of bone chips could be a new safe source of human type I collagen.

Culturing human CECs is not an easy task since most of the corneas are used for tissue transplant and those that are discarded have a limited cell density. During corneal transplant, peripheral Descemet’s membrane is usually discarded and only its central button is used for graft applications. The unused endothelial tissue may be used in regenerative medicine for the development of a tissue-engineered endothelium since they come from an optimal cell source.

The aim of this study was the development of a new approach for endothelial tissue engineering using products normally processed in tissue banks: remnants of human cancellous bone chips and Descemet’s peripheral rings of corneas previously used for PK. With this approach we were able to produce an artificial corneal endothelial graft, using human purified type I collagen membranes (HPCM) with confluent human and rabbit CECs expressing characteristic corneal endothelial markers. Finally, this artificial lamellar endothelium was grafted in an experimental rabbit model to restore an endothelial dysfunction, leading to complete recovery of corneal transparency and thickness at 6 weeks.

## Materials and Methods

### Donors

Human tissue was handled according to the Declaration of Helsinki. Corneal tissue and fresh human cancellous bone were obtained, stored and processed at the Asturias regional tissue bank (Centro Comunitario de Sangre y Tejidos, Oviedo, Asturias, Spain) according to Spanish laws.

### Collagen type I isolation and quantification

Collagen isolation was performed as previously described [36]. Briefly, 10 cancellous bone chips samples (between 5 and 15cm^3^ of bone) were received from the local tissue bank and stored at -80°C until use.

Cancellous bone chips were milled and demineralized using an adaptation of a previously reported method [37]. In brief, the granules were demineralized under agitation (300rpm) in 0.5N HCl (25ml/g of bone) at room temperature for 24h. After demineralization the resultant material was rinsed with distilled water. The lipids in the demineralized powder were then rinsed out under agitation with a 1:1 mixture of chloroform and methanol for 1h at room temperature and then repeatedly rinsed, first in methanol and then, in distilled water. The material was then freeze-dried in a Lyoquest -85° Eco (Telstar, Tokyo, Japan) and stored at -80°C until required.

A previously reported pepsin digestion and solubilization technique [38] was employed for the isolation of type I collagen. Lyophilized samples were digested in a 100ml/g 0.01N HCl solution containing 1mg/ml pepsin (Sigma-Aldrich, MO, USA). The suspension was agitated at room temperature for 96h, centrifuged at 10,000xg for 30 minutes, the precipitate discarded, and then aliquoted and stored at -80°C until required.

The collagen concentration was determined using a hydroxyproline assay kit (Sigma-Aldrich, MO, USA). The hydroxyproline content of pepsin digested collagen was determined by the oxidized hydroxyproline with 4-(Dimethylamino)benzaldehyde (DMAB) reaction, which results in a colorimetric (560nm) product, proportional to the hydroxyproline present. The total collagen content (mg/ml) of the digests was determined using the relationship that hydroxyproline forms 14% of total collagen [39].

### Gel electrophoresis and western-blot

Electrophoresis was carried out as described by Laemmli [40], using 8% separating gels, which were 1.5mm thick. Samples containing 20.7μg purified collagen and human commercial collagen (Millipore, MA, USA) as control were boiled at 100°C for 5 minutes prior to gel loading. Gels were electrophoresed at 100V (constant voltage) followed by transfer onto a polyvinylidene fluoridemembrane (PVDF, 120V, 40 minutes). After transfer, PDVF membranes were immunoblotted with anti-type I collagen antibody (1:200), and 10% normal goat serum (Abcam, Cambridge, UK) as a blocking agent, overnight at 4°C. Proteins were visualized using a Fusion FX7 chemiluminiscence detection system (VilberLourmat, Torcy, France).

### Collagen purity assessment

For purity assessment, six random samples were analyzed by liquid chromatography–mass spectrometry (LC/MS).

Samples were incubated at room temperature with ammonium bicarbonate (50mM, final volume of 50μl) in 8M urea. After 30 minutes 4μl of ditiothreitol (DTT, 200mM in 50mM ammonium bicarbonate) was added and incubated at room temperature for 60 minutes, followed by incubation in iodoacetamide (IA, 50mM in 50mM ammonium bicarbonate, 5μl) for another 60 minutes in the dark. Excess of IA was quenched by adding 20μl DTT (200mM in 50mM ammonium bicarbonate). Finally, samples were diluted up to a concentration of 1.5M urea, and incubated with trypsin (12.5μg/ml in 50mM ammonium bicarbonate) overnight at 37°C. After digestion, samples were dried out in a RVC2 25 speedvac concentrator (Christ, Osterode am Harz, Germany).

LC was performed using an NanoAcquity nano-HPLC (Waters, MA, USA), equipped with a Waters BEH C18 nano-column (200mm x 75μm ID, 1.8μm), a chromatographic ramp of 30 minutes (5 to 60% ACN) was used with a flow rate of 300nl/minute. Mobile phase A was water containing 0.1% v/v formic acid, while mobile phase B was CAN containing 0.1% v/v formic acid. A lock mass compound [Glu1]-Fibrinopeptide B (100fmol/μl) was delivered by an auxiliary pump of the LC system at 500nl/minute to the reference sprayer of the NanoLockSpray (Waters, MA, USA) source of the mass spectrometer. 0.5μg of each sample were loaded for each run.

MS was performed using a Synapt G2Si ESI Q-Mobility-TOF spectrometer (Waters, MA, USA) equipped with an ion mobility chamber (T-Wave-IMS) for high definition data acquisition analyses. All analyses were performed in positive mode ESI. Data were post-acquisition lock mass corrected using the double charged monoisotopic ion of [Glu1]-Fibrinopeptide B. Accurate mass LC-MS data were collected in HDDA mode that enhances signal intensities using the ion mobility separation step.

Database searching was performed using MASCOT 2.2.07 (Matrixscience, London, UK) against a UNIPROT—Swissprot database filled only with entries corresponding to *Homo sapiens* (without isoforms). For protein identification the following parameters were adopted: carbamidomethylation of cysteines as fixed modification and oxidation of methionines, and hydroxylation as variable modifications, 10ppm of peptide mass tolerance, 0.5Da fragment mass tolerance and up to 3 missed cleavage points, Peptide charges of +2 and +3.

Content of purified proteins was quantified using the Exponentially Modified Protein Abundance Index (emPAI) as previously described [41]. The protein contents in molar fraction percentages (mol%) were calculated according to the following equation:

$$mol\%=\left[\frac{emPAI}{\Sigma \left(emPAI\right)}\right]\;100$$

The identified proteins were categorized into three groups: type I collagen, other collagenous proteins and non-collagenous proteins.

### Preparation of the collagen membranes

An amount of 3mg/cm^2^ isolated type I collagen was casted into a silicone mold and air-dried at room temperature. To improve their resistance, HPCM were crosslinked according to modified existing protocols [42]. Briefly, ultraviolet light (UV) irradiation was conducted by placing HPCM in a glass dish into a BLX-E254 254nm UV irradiation lamp (VilberLourmat, Torcy, France) exposed to a UV intensity of 3.19mW/cm^2^/s during 100 minutes.

### Optical analysis and mechanical testing

The entire tests were carried out at 20±0.1°C and before starting any measurements, the samples rested for at least 5 minutes, allowing the stresses induced during samples load to relax. This study was performed employing pre-wet (1X PBS for 24h at room temperature) 10mm diameter HPCM. All tests were carried out at least in duplicate.

Light transmission measurements were made using a narrow spectral region between 400 and 700nm using a SPELEC Spectroelectrochemical Instrument (Dropsens, Asturias, Spain) equipped with a Deuterium 215-400nm and Tungsten Halogen 360–2,500nm light source and a linear silicon CCD array with a detection range 200-900nm detector. A human cornea (74 years old) was employed as a control.

The crosslinked and non-crosslinked HPCM samples were mechanically tested with a TA.TXplus (Stable Micro Systems) texturometer with a 5mm diameter SMS P/5 S spherical probe in order to obtain the burst strength (g) and the distance at burst (mm) of the HPCM.

Statistical analyses were performed using IBM SPSS software. Significant differences among defined groups were tested using the t test. A difference level of p<0.05 was considered as statistically significant.

### Isolation and culture of corneal endothelial cells on HPCM

Corneal endothelial cells were cultured in Optimem I (Life Technologies, CA, USA) supplemented with 8%v/v FBS, 20μg/ml ascorbic acid 2-phosphate, 0.08% chondroitin sulfate, 200mg/l calcium chloride, 10U/ml penicillin and 10 μg/ml streptomycin (Sigma-Aldrich, MO, USA) and 5ng/ml epidermal growth factor (Austral Biologicals, CA, USA).

Human CECs were obtained from 26 peripheral endothelial rings from corneas previously used for PK which were maintained in Eusol-C storage medium (Alchimia, Ponte S. Nicolò, Italy) at 4°C for less than 10 days before use. The mean age of corneal donors was 63±2.17 years old and the endothelial cell density, determined prior to the surgery using a Cellcheck specular endothelial microscope (Konan Medical, USA), was 2,653±82.51 cells/mm^2^. Descemet´s membrane along with endothelial cells were carefully peeled off under a dissecting stereomicroscope following the Schwalbe line, and the peripheral endothelial ring was maintained overnight at 37°C in a culture plate (2cm^2^), previously treated with FNC coating Mix^®^ (Athena Enviromental Sciences, MD, USA), with 1ml culture medium. The following day, the excess medium was removed to a volume of 150μl and then, the peripheral endothelial ring was placed as an explant in the culture plate.

Rabbit corneal endothelial cells (rabbit CECs) were isolated from healthy New Zealand white male rabbits (2 months and 2.0–2.5kg in body weight) obtained from the Animal Housing of the University of Oviedo (Oviedo, Asturias, Spain). All animals were treated in accordance with the Association for Research in Vision and Ophthalmology (ARVO) statement for use of animals in ophthalmic and vision researches. The protocols were approved (PROAE 24/2016) by the Committee on the Ethics of Animal Experiments of the University of Oviedo and the Animal Production and Health Service of Asturias. Rabbits were kept under a 12/12 day/night light cycle with food and water ad libitum and were monitored on a daily basis.

Descemet´s membrane along with endothelial cells were carefully peeled and digested with trypsin/EDTA 0.25% (Sigma-Aldrich, MO, USA) for 30 minutes at 37°C. After that, the trypsin was neutralized with culture medium. The loosened cells were centrifuged using an Eppendorf 5702R centrifuge (Eppendorf, Hamburg, Germany) at 0.4rcf for 10 minutes and the supernatant was removed. Fresh medium was added and the cells were seeded on a culture plate previously treated with FNC coating Mix^®^.

When human or rabbit cultures were confluent, cells were digested with accutase (Sigma-Aldrich, MO, USA). The loosened cells were centrifuged using an Eppendorf 5702R centrifuge at 0.4rcf for 10 minutes and the supernatant was removed. Fresh medium was added and the cells were seeded onto a HPCM using a device (11mm diameter) [43] that facilitates handling, cell culture and transport of the membranes.

All cells were cultivated under the same conditions (humidified atmosphere at 37°C, 5% CO_2_, medium changed 3 times per week).

### Examination of cell cultures

Cellular growth was assessed using a Leica DMIL LED phase contrast microscope (Leica, Wetzlar, Germany); photos were taken with an attached EC3 camera (Leica, Wetzlar, Germany).

Confluent cultured HPCM were fixed using ice-cold methanol for 10 minutes for their analysis by phase contrast microscopy, scanning electron microscopy (SEM) and immunocytochemistry. Methanol fixed cultured HPCM were divided in two parts, one half was rinsed with PBS solution twice for 10 minutes and permeabilized in a PBS solution containing 0.3% Triton-X100 for another 10 minutes. Next, the samples were incubated with primary antibody containing 10% normal goat serum (Abcam, Cambridge, UK) at 4°C overnight. Mouse Na^+^/K^+^ ATPase (Millipore, MA, USA) (1:100), rabbit zonula ocluddens-1 (ZO-1) (Life Technologies, CA, USA) (1:100) and rabbit type IV collagen (Abcam, Cambridge, UK) (1:100) single immunostains were performed in order to confirm their phenotype. Subsequently, the samples were incubated with corresponding secondary antibody (Life Technologies, CA, USA) (1:500) for 2h at room temperature. Between incubations samples were washed 3 times with PBS for 10 minutes. Immunolabeled cells were stained with DAPI to allow nuclei visualization.

The other half was cryoprotected in 30% sucrose at 4°C for 24h and frozen embedded in Tissue-Tek^®^ O.C.T.^TM^ Compound (Sakura Finitek Europe B.V., Zoeterwoude, The Netherlands). Next, 10μm sections were cut with a cryostat (Leica, Nussloch, Germany) and placed on commercially treated slides (Fisher-Scientific, PA, USA). Immunostaining was performed as described in the previous section using antibodies against ZO-1, Na^+^/K^+^ ATPase and type IV collagen.

All the samples were critically examined in a Leica DM6000B fluorescence microscope (Leica, Wetzlar, Germany).

Surface morphology of cultured and non-cultured HPCM was examined by SEM. HPCM fixed in ice-cold methanol were rinsed in PBS, followed by dehydration through a graded series of acetone (30%, 50%, 70%, 90% and 100%) for 10 minutes respectively and dried by the critical-point method. Afterwards, they were coated with gold under vacuum and observed with a JEOL 6610LV scanning electron microscope, (JEOL co., Tokyo, Japan) at 20kV accelerated voltage.

### Transplantation of HPCM in a rabbit model

DMEK surgery was performed in six New Zealand white rabbit. Rabbits were divided into three groups: HPCM with or without cultured endothelial cells and rabbits with only peeled off Descemet’s membrane.

**Fig 1:**
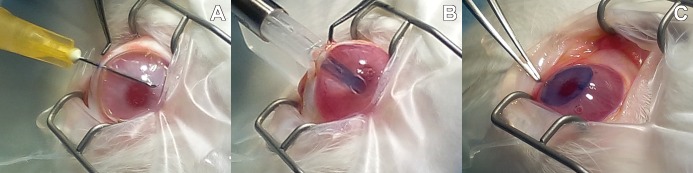
DMEK surgical procedure for HPCM transplantation. (A) Descemetorrexis from the posterior stroma, (B) injected grafts of HPCM with or without RCECs and (C) unfolded HPCM inside the anterior chamber.

Animals were anesthetized with buprenophine and meloxicam, and then intubated and ventilated with isofluorane 2%. After topical administration of double anesthetic colicursi (tetracaine 0.1% and oxybuprocaine 0.4%), a 4mm wound incision was made at the limit of the corneoescleral tissue of each right eye with a slit knife, and the corneal endothelium was removed from the anterior chamber with a 30-gauge needle. HPCM with or without rabbit CECs were cut using an 8.5mm diameter trephine and stained with trypan blue solution. Grafts of HPCM, as a half-rolled taco, were placed in a disposable inserting set for Descemet lenticle with a viscoelastic agent (E.Janach, Como, Italy), injected and unfolded into the anterior chamber and fixed to the posterior stroma stripped of Descemet´s membrane (Fig 1). The sclerocorneal wound was closed with nylon sutures and a subconjunctival dose of Trigon Depot (triamcinolone acetonide 40mg/ml) was administered.

After transplantation, rabbits were treated with Tobradex (dexamethasone 1mg/ml + tobramycin 3mg/ml) and Timabak (timolol 2.5mg/ml) eye drops twice a day during all the follow-up period. The exterior appearance of rabbit eyes was monitored by taking photographs at the day of surgery, 24h after surgery, and once a week for the duration of the experiment. Six weeks after transplantation, corneal thickness was measured by anterior segment optical coherence tomography (AS-OCT) using a OCT CASIA SS-100 (Tomey, Erlangen, Germany). Finally, rabbits were euthanized by an intravenous overdose of pentobarbital sodium.

Corneas were excised, rinsed with PBS solution and fixed using ice-cold methanol for 4h. Corneal tissues were embedded in paraffin and then, hematoxylin-eosin (H-E) stain and immunostaining against ZO-1 and Na^+^/K^+^ ATPase was performed as described in the previous section.

## Results

### Assessment of collagen content

The rate of type I collagen extraction was calculated from 10 independent isolations using different human cancellous bones. The mean soluble collagen contents were determined to be 0.23mg of collagen per mg of initial dry weight (Table 1). The mean concentration of the soluble collagens was 2.46mg/ml. For each membrane, we used about 10mg collagen that allows the preparation of at least 5 HPCM if employing the poorest collagen isolation.

**Table 1:** Assessment of collagen content (mg) and collagen concentration (mg/ml).

Samples	Dry weight (mg)	Collagen isolation (ml)	Collagen concentration (mg/ml)	Total purified collagen (mg)
**1**	1,753	165	2.92	481.80
**2**	2,005	110	1.47	161.70
**3**	378	54	4.53	244.62
**4**	5,608	527	1.63	859.01
**5**	202	17	3.10	52.70
**6**	1,266	110	2.10	231.00
**7**	2,145	175	2.24	392.00
**8**	2,383	198	1.62	320.76
**9**	754	49	2.00	98.00
**10**	634	47.50	2.98	141.55

### Gel electrophoresis and western blot

**Fig 2:**
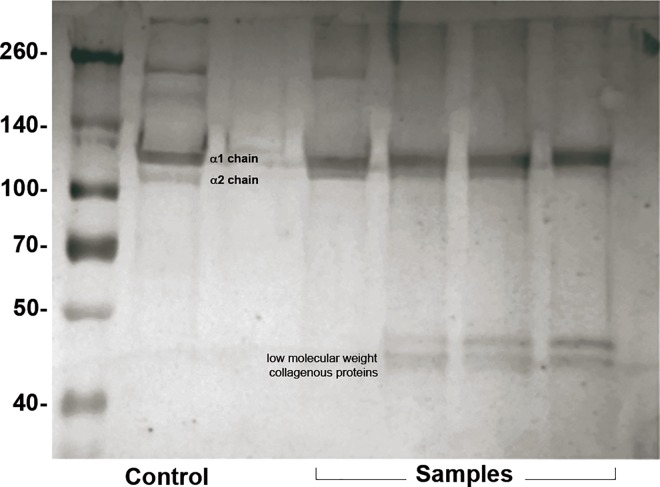
Analysis by SDS-PAGE and immunobloting of type I collagen preparation from human cancellous bone. Control group: lane 1; Samples: lanes 2, 3, 4 and 5.

The acid extract of human cancellous bone was subjected to SDS-8% acrylamide gel electrophoresis (Fig 2) and immunoblotting with polyclonal rabbit antibody to type I collagen (Millipore, MA, USA). The SDS-PAGE band pattern of the purified material contained at least two different α chains, but the separation of these two chains was not as pronounced as in the control group. Moreover, ß chain was also found in some of the samples as well as some low molecular weight bands.

### Collagen purity assessment

LC/MS analysis and posterior emPAI quantification are shown in Table 2. Type I collagen protein was present as a major product in all the purified samples (α1 = 64.35±1.86%mol; α2 = 34.09±1.79%mol). Other collagenous proteins (type II, III, IV, V, XI and XII) were also found as purification subproducts. Finally, residual contaminant proteins such as actin or type I and type II keratins, were found in a 0.80±0.41%mol.

**Table 2:** %mol of proteins determined by LC/MS.

Samples	Type I collagen		Other collagenous proteins						Non-collagenous proteins
	α1	α2	Type II	Type III	Type IV	Type V	Type XI	Type XII	
**1**	64.18	35.44	0.09 (α1)	0.09 (α1)	0.04 (α5)	-	-	-	0.15
**2**	69.3	28.64	0.32 (α1)	0.61 (α1)	-	0.06 (α1)	0.03 (α1) 0.03 (α2)	-	0.95
**3**	58.73	37.21	0.39 (α1)	0.83 (α1)	-	-	-	0.06 (α1)	2.78
**4**	68.18	30.56	0.19 (α1)	0.45 (α1)	0.03 (α5)	0.08 (α1) 0.08 (α2)	0.03 (α1) 0.03 (α2)	-	0.39
**5**	66.62	32.35	0.08 (α1)	0.38 (α1)	0.04 (α5)	0.08 (α1)	0.04 (α1)	-	0.36
**6**	59.10	40.32	0.14 (α1)	0.07 (α1)	0.01 (α5)	0.06 (α1) 0.05 (α2)	0.01 (α1) 0.01 (α2)	-	0.19

#### Preparation of the HPCM

**Fig 3:**
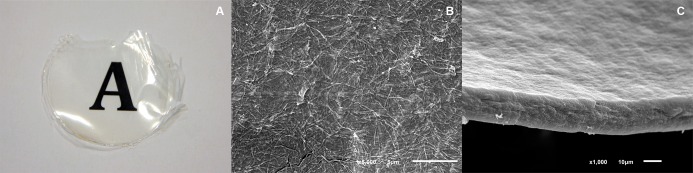
(A) Macrophotograph appearance of HPCM, (B) SEM microphotograph displaying fibrillary ultrastructure and (C) thickness of HPCM.

HPCM were obtained by evaporating the soluble collagen content of the purified extract and crosslinking by UV light exposure. Fabricated membranes had an average area of 3.46cm^2^ and thickness of 20μm showing a randomly oriented fibrillar structure when the surface was observed under SEM (Fig 3).

### Optical analysis and mechanical testing

**Fig 4:**
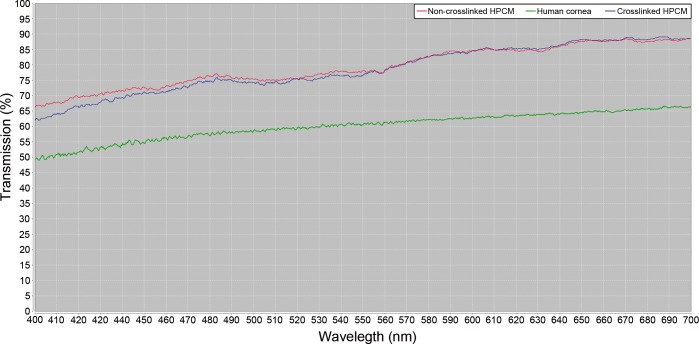
Optical properties (% light transmission) of crosslinked, non-crosskinked HPCM and human control cornea.

As shown in Fig 4, HPCM was optically transparent at all wavelengths of the visible light electromagnetic spectrum with a mean value of light transmission of 78.10±1.77% and 77.89±1.43% in crosslinked and non-crosslinked HPCM respectively, showing no significant differences at any point of the visible light electromagnetic spectrum. When these values were compared with the light transmission of a control human cornea (67.84±2.51), crosslinked and non-crosslinked HPCM displayed statistically significantly higher values (p<0.01).

Mechanical testing revealed an enhancement in both: the resistance, determined by the burst strength, and the elasticity, determined by the distance at burst in the crosslinked HPCM. This difference (p<0.01) was statistically significant compared to the non-crosslinked HPCM (Table 3).

**Table 3:** Mechanical properties of HPCM calculated using TA.TXplus texturometer (values are shown as mean±SEM).

	Crosslinked membranes	Non-crosslinked membranes
Burst strength (g)	178.12±19.46	19.78±7.24
Distance at burst (mm)	2.53±0.27	0.78±0.33

### Isolation, culture and examination of corneal endothelial cells on HPCM

**Fig 5:**
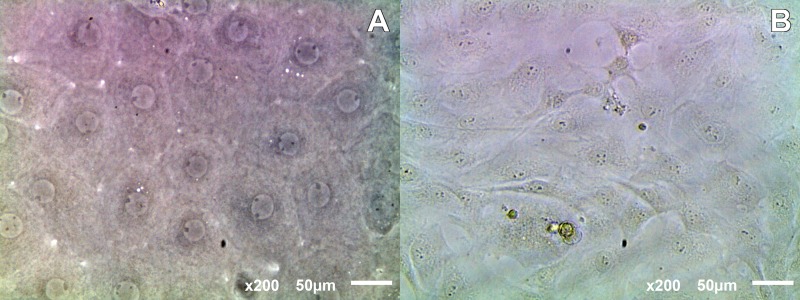
Microphotographs of human CEC displaying hexagonal and endothelial-mesenchymal transformed morphology.

Human CECs were observed to have migrated from the explant onto the plate after 3–4 days in culture, and by day 30 a monolayer of compact cells had formed on the plate, immediately adjacent to the explant in 13 of the 26 peripheral endothelial rings processed. These cell displayed their typical hexagonal endothelial morphology, while in the other 13 cultures, cells showed signs of endothelial-mesenchymal transition resulting in the loss of corneal endothelial specific hexagonal morphology and transformation into an elongated and fibroblast like abnormal phenotype (Fig 5). No significant differences were found between different endothelial cultures when comparing cell density, determined prior to the surgery, or age of the corneal donors (Table 4). After 30 days, confluent hexagonal endothelial cells were detached and subcultured on HPCM. In the same way, rabbit CECs were able to attach and proliferate when isolated, by trypsin/EDTA digestion, and subcultured on HPCM.

**Table 4:** Human CEC cultures displaying typical hexagonal cell morphology or endothelial-mesenchymal transformation grouped by endothelial cell density, determined prior to surgery, and age of the corneal donors (values are shown as mean±SEM).

	Age (years)	Cellular density (cell/mm^2^)
Typical hexagonal cell morphology	64.33±2.69	2,546±121.8
Endothelial-mesenchymal transformation	61.91±3.57	2,760±107

**Fig 6:**
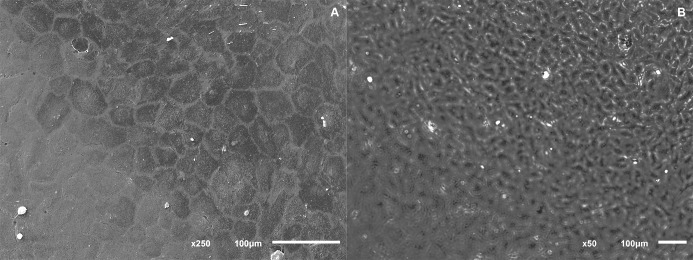
(A) SEM and (B) phase contrast microscopy micrographs of human CECs growing on HPCM.

Under phase contrast microscope and SEM, human CECs and rabbit CECs demonstrated their ability to attach and proliferate when cultured on HPCM, maintaining their polygonal morphology (Fig 6).

**Fig 7:**
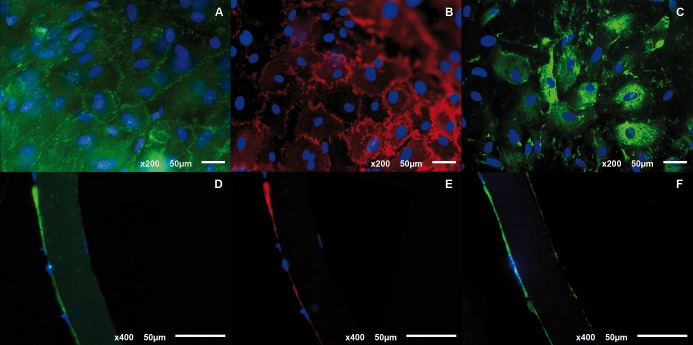
**(A, B&C) Whole-mount and (D, E&F) frozen section immunostains of human CEC cultured on HPCM.** ZO-1 (A&D), Na^+^/K^+^ ATPase (B&E) and type IV collagen (C&F). Nuclei stained in blue.

**Fig 8:**
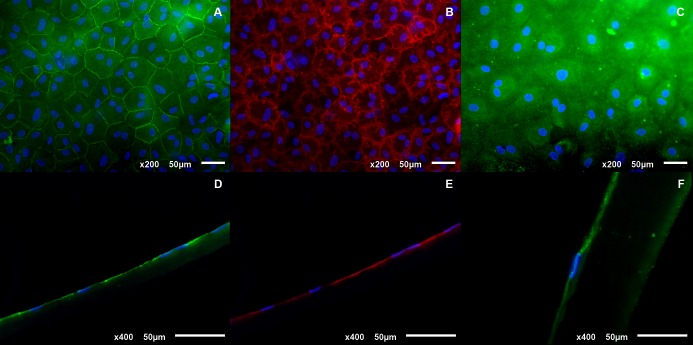
**(A, B&C) Whole-mount and (D, E&F) frozen section immunostains of rabbit CEC cultured on HPCM.** ZO-1 (A&D), Na^+^/K^+^ ATPase (B&E) and type IV collagen (C&F). Nuclei stained in blue.

Immunohistochemical analysis revealed a positive stain for ZO-1, a tight junction associated protein responsible for establishing the passive permeability properties of the endothelial barrier and Na^+^/K^+^ ATPase, an integral membrane protein responsible for regulating pump functions. Additionally, it also showed a positive stain for a newly formed basal membrane protein, type IV collagen (Figs 7 and 8).

### Transplantation of HPCM

**Fig 9:**
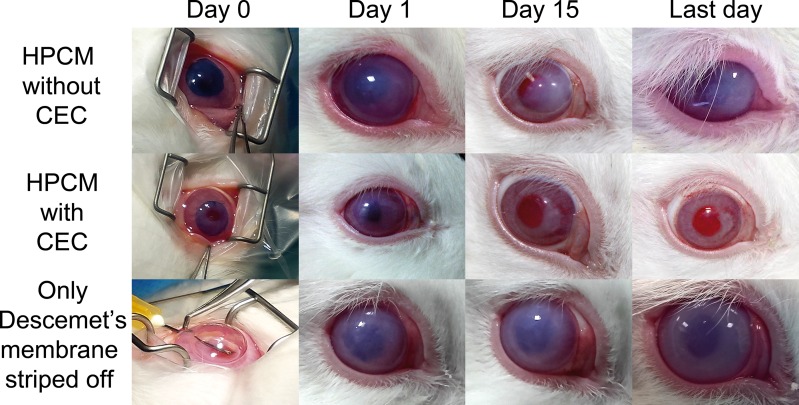
Macrophotographs of the exterior appearance of transplanted rabbit eyes during the 6 week follow-up period.

Corneal edema and white turbidity appeared few days after surgery in the three groups, rabbits transplanted with or without rabbit CECs cultured on HPCM and rabbits with only Descemet’s membrane peeled (Fig 9). Transplanted corneas with cultured rabbit CECs on HPCM began to become transparent by day 10 and corneal transparency was maintained up to 6 weeks. However, the eyes in the group without rabbit CECs on HPCM and rabbits with peeled off Descemet’s membrane did not restore corneal transparency and retained a corneal edema during the follow up period. No obvious signs of immune rejection were found in any group.

**Fig 10:**
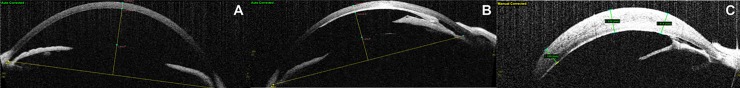
AS-OCT of transplanted rabbit corneas displaying corneal thickness at 6 weeks.

Results of AS-OCT revealed HPCM with cultured endothelial cells as a fully integrated component in the corneal tissue, displaying a similar corneal thickness when compared to its healthy contralateral cornea. On the other hand, HPCM without corneal endothelial cells and rabbits with peeled off Descemet’s membrane only revealed an enhancement in corneal thickness reflecting a loss of corneal endothelial functionality (Fig 10).

**Fig 11:**
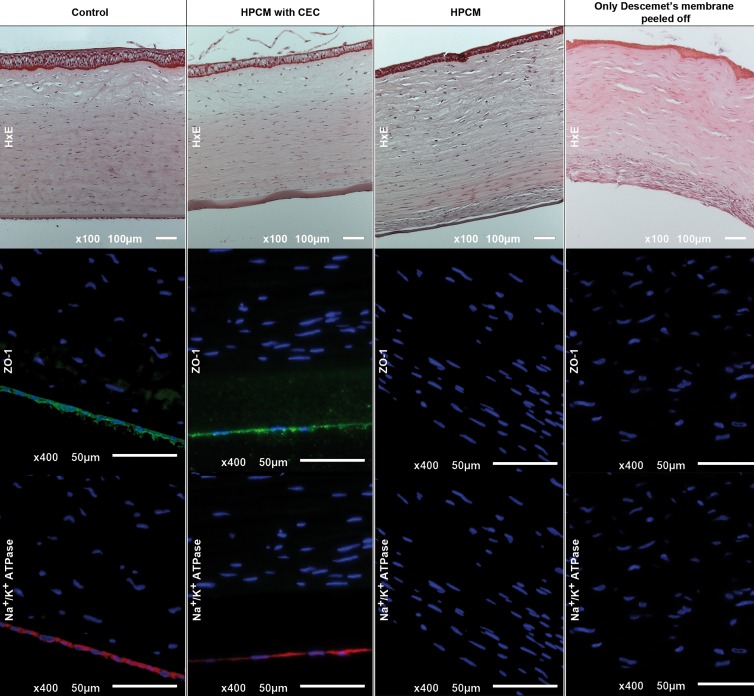
Histological analysis of transplanted rabbit corneas.

The histological analysis showed a slightly marked fibrotic tissue in the posterior segment of the stroma in rabbits transplanted without corneal endothelial cells on HPCM and an apparent corneal edema and fibrotic tissue in the rabbits with peeled off Descemet’s membrane. HPCM, in rabbits transplanted with cultured cells, was attached tightly to the corneal stroma and rabbit CECs formed a continuous monolayer with the same morphology and phenotypical markers, ZO-1 and Na^+^/K^+^ ATPase, as a healthy control eye (Fig 11). No sign of endothelia remains were found in any of the transplanted corneas.

Hematoxilin-eosin stain (top row), ZO-1 (middle row) and Na^+^/K^+^ ATPase (bottom row) immunofluorescence of: control healthy cornea (first column), transplanted HPCM with (second column) or without (third column) rabbit CECs and cornea with only peeled off Descemet’s membrane (fourth column) at 6 weeks. Nuclei stained in blue.

## Discussion

Human corneal endothelial cellular loss or damage leads to stromal edema, loss of transparency, and will eventually lead to blindness, requiring a healthy endothelial layer to reverse the edema [44]. Nowadays the lack of corneal tissue is one of the hardest hurdles to overcome for corneal repair. This barrier is even more pronounced in countries with a poor donation system or in those whose ethics or religious convictions forbid tissue and organ donations.

Cell therapies and tissue engineering may be the future tools that allows these limitations to be surpassed through the optimization of protocols for the *in vitro* expansion of human CECs and the development of tissue-engineered scaffolds.

In recent years, the culture technics of human CECs have been extensively improved allowing their isolation and expansion in culture [45,46]. A large number of studies have demonstrated the possibility of transplanting them in animal models with [25] or without [47] a carrier. Intracameral injection of CECs for corneal endothelial dysfunctions has appeared as a promising therapy, even more so if it is associated with the use of ROCK kinase inhibitor to enhance the attachment of injected CECs [3,48]. However, these new approaches will have to overcome the problems associated with injection of CECs, such as systemic dispersion of CECs. At the moment, the use of a scaffold that allows the growth of CECs appears as a better way to control the corneal attachment of CECs. However, future studies will need to elucidate the most effective treatment for corneal endothelial dysfunctions.

Different scaffolds have already been used as artificial Descemet’s membrane for the growth of human CECs. Several groups have used bovine or porcine type I collagen as scaffold for these purposes, showing in different animal models that corneal endothelial dysfunctions can be treated [25,30].

However, the clinical application of tissue engineered therapies should avoid the use of xenogeneic products as far as possible. For this purpose, we have developed an artificial endothelial graft using type I collagen isolated from remnant human cancellous bone.

The analysis of emPAI based molar fractions of our samples demonstrate that type I collagen can be isolated in a relatively easy, short and reproducible process (with a low presence of different bone associated collagens and residual contaminants) from cancellous bones routinely processed in tissue banks. In our local tissue bank 8,000cc of cancellous bone are processed every year and, in many donors, small tissue remnants (4-10cc) are left unused. These cancellous bone remnants could potentially be used for type I collagen isolation.

Moreover, this product is safe because the donors are selected based on their clinical history and serological tests (HIV; HVB; HCV and syphilis) and its origin is traceable because tissue banks stores the donor´s data.

In the present study, and using the isolated collagen, we developed a HPCM with a thickness similar to Descemet´s membrane. These HPCM needed to be crosslinked by UV light to increase their mechanical properties (resistance and elasticity), however, light transmission values weren’t affected and HPCM still showed better optical qualities than a normal human cornea.

On the other hand, we cultured human CECs from peripheral endothelium of corneas previously used as grafts for PK; peripheral ring cells have more regenerative capacity [49] and are a source of cells that are not used in transplant procedures. Per peripheral ring and with the culture method here described, we are able to obtain a HPCM of 11mm in diameter cultured with polygonal human CECs in one out of two corneas processed. This was not related to the age nor the endothelial cellular density of corneal donors, suggesting, as previously described [50], a direct relationship between the relative health of the donors before death as well as the period between death, tissue processing and cellular culture in the ability of these cells to grow and its outcome in culture. HPCM have proven to be a good substrate for human CECs since cells growing on HPCM expressed characteristic markers, such as ZO-1 and Na^+^/K^+^ ATPase. Moreover, they also expressed type IV collagen, a protein present in the Descemet’s membrane, displaying the same expression pattern as that found in a healthy corneal endothelium.

To evaluate *in vivo* application of our artificial endothelial grafts, we performed a DMEK surgery in a rabbit model. After surgery, no signs of immunological rejection were found, and the HPCM with cultured rabbit CECs were able to restore corneal transparency in the transplanted corneas, appearing as a fully integrated lamellar graft in H-E stain with a normal corneal thickness as shown by AS-OCT. Moreover, not finding any sign of endothelium remains indicates that a complete descemetorrexis was performed. This implies that the restoration of corneal transparency did not originate from autologous cell proliferation. Additionally, immunofluorescence results indicate that rabbit CECs formed a continuous monolayer with the same phenotypical markers that in a control cornea.

Nevertheless, our current work scheme still has some issues to resolve. We are currently using a commercially available porcine derived pepsin in the extraction process, because this protease produces a better yield of collagen extraction. Moreover, we use FBS for the culture of human CECs. In future works, it is desirable that xenogeneic products were replace by human derived products.

In conclusion, our work shows that it is possible to generate new artificial lamellar endothelial grafts using human type I collagen and endothelial cells from remnant tissues from tissue banks and that these artificial lamellar endothelial grafts can be transplanted in a rabbit model with satisfactory results. With this new approach it could be possible to increase the total number of patients grafted by generating new artificial endothelial lamellar grafts.
